# Requirements specification, design, and evaluation of dental image exchange and management system with user‐centered approach: A case study in Iran

**DOI:** 10.1002/hsr2.1760

**Published:** 2023-12-17

**Authors:** Bahlol Rahimi, Sajjad Karimian, Aisan Ghaznavi, Mohammad Jafari Heydarlou

**Affiliations:** ^1^ Health and Biomedical Informatics Research Center Urmia University of Medical Sciences Urmia Iran; ^2^ Student Research Committee Urmia University of Medical Sciences Urmia Iran; ^3^ Department of Oral and Maxillofacial Radiology, School of Dentistry Urmia University of Medical Sciences Urmia Iran; ^4^ Department of Oral and Maxillofacial Disease, School of Dentistry Urmia University of Medical Sciences Urmia Iran

**Keywords:** health information systems, interactive exchange radiology image system, program evaluation, radiology information systems, requirements specification, software design

## Abstract

**Background and Aims:**

Systems existing in hospital or clinic settings offer services within the physical environment. Examples of such systems include picture archiving and communication systems, which provide remote services for patients. To develop a successful system, methods like software development life cycles (SDLCs) and design techniques, such as prototyping, are needed. This study aimed to specify requirements, design, and evaluation of dental image exchange and management system using a user‐centered approach.

**Methods:**

This cross‐sectional study was conducted in four phases, each corresponding to different stages of SDLCs. User‐needs data were used to gathered by interviews and observations. A prototype was developed using object‐oriented programming and presented to users for feedback. Finally, focus group was used to finalized the prototype into the desired system.

**Results:**

User needs were identified and prioritized from the outset, with ease of use, security, and mobile apps being their most essential requirements. The prototype underwent several iterations of design and evaluation in focus group sessions until users were satisfied, and their feedback was incorporated. Eventually, the prototype was refined into the final system with users' consent.

**Conclusion:**

The study revealed that instant access to information, voluntary participation, user interface (UI) design, and usefulness were critical variables for users and should be integral to any system. Successful implementation of such a system requires careful consideration of end‐users' needs and their application to the system. Moreover, integrating the system with electronic health records can further enhance the treatment process and the efficiency of medical staff. The voluntary perspective of users played a significant role in achieving an exemplary UI and overall satisfaction with the system. Developers and policymakers should consider these aspects in similar system development projects.

## 1 BACKGROUND AND AIMS

In health‐related contexts, systems like hospital information systems (HIS), picture archiving and communication systems (PACS), and radiology information systems (RIS), primarily serve patients and healthcare providers within physical locations like hospitals or clinics.^1^, ^2^ RIS and PACS systems, for instance, allow the transmission of textual and visual patient data through web services in healthcare settings.^3^, ^4^ Despite the advantages of web‐based image systems, heir development faces limitations such as privacy, security, cost‐related issues, usability, evaluation, infrastructure, and hardware accessibility. Additionally, with the prevalence of mobile phones, network connections are available to all users, including patients and physicians.^3^, ^5^, ^6^, ^7^ To ensure the success of these systems, comprehensive evaluation and validation are necessary. This involves assessing functionality, cost analysis, and achieving the system's intended goals.^8^ A key factor in the success of any system is the consideration of the needs of all users during the design and implementation process. Neglecting this aspect may lead to significant failures,^4^, ^7^ making proper evaluation even more crucial.^5^, ^6^, ^8^ Various methods are available for the successful system development.^1^

In general, the failure of an electronic health record (EHR) system or any related health contextual systems can be attributed to a lack of attention to users' needs.^9^ Indeed, involving users in the early stages of prototype design is a key factor contributing to the success of a system.^10^ Several studies have highlighted the significance of focusing on users and their needs for the successful implementation of a system.^11^, ^12^, ^13^ With the increasing use of medical information exchange systems, the importance of users' feedback in the design, implementation, and acceptance of these systems has become highly significant.^14^ To develop effective information systems, system developers, analyzers, and designers should utilize various methods, including waterfall methods, prototype development, and other approaches. Proper development and evaluation of the working system are essential to achieve the desired outcomes.^5^, ^6^, ^8^

Appropriate design and creation in software or system development are guided by the software development life cycle. This method involves several stages, including designing, planning, programming, implementing, and maintaining the required system or software. It provides guidance to the management and executive team throughout the process, contributing to the success and advantages of the developed systems. Evaluating the needs of users, especially end‐users, is crucial in this process and is typically undertaken by system designers and programmers.^13^

Human–computer interaction is an effective method for successful information systems analysis. It focuses on user‐affected factors, such as behavioral, cognitive, ease of use, and ergonomic considerations. The “User‐Centric Approach” is one of the approaches used in software analysis ^5^, ^6^, ^7^, ^15^ which emphasizes considering affairs from the users' perspective. Understanding how users will interact with the system based on their different approaches is a critical aspect of system analysis. Gathering information that meets the complex needs and expectations of all stakeholders and system end‐users is essential for creating an effective system that aligns with existing processes.^5^, ^6^, ^7^, ^15^, ^16^

The purpose of this study is to specify the requirements, design, and evaluation of a dental image exchange and management system using a user‐centered approach. The development of this system involved the optimal utilization of available resources. Additionally, the study aimed to identify factors affecting stakeholder efficiency (e.g., dentists, patients, and assistants) and data management within the oral and maxillofacial radiology images context in Urmia's private sector dentistry clinics. Notably, these clinics lacked any available system for exchanging patients' data prior to this study.

## 2 METHODS

The current situation in dentistry clinics studied for exchanging radiology images is traditional. Patients visit the dentist, receive an X‐ray order, and then go to a dental imaging center where X‐ray images are taken and printed. After obtaining the images, they return to their doctors to show the results. In the past, image sharing was possible through certain social messengers, but these platforms encountered issues and are no longer operational. Currently, dental images are sent to the dentist either in hard copy or soft copy, like a CD, without any information exchange between the private sector and the Iranian government. By developing such systems, we aim to facilitate information exchange and enhance access to comprehensive EHR systems. Presently, the Iranian EHR system, known as “SEPAS,” only contains demographic and insurance information in its initial stage of development, and it gathers data solely from hospitals. However, future plans aim to include all patient information to create a comprehensive EHR system.

The present system was designed and developed iteratively, based on prototype development. In the first phase, users' requirements were gathered through ethnographic methods such as interviews and observations to design and develop the necessary system. Interviews were conducted to collect information on system features, user interface (UI), user needs, and expectations. Observations were utilized to understand users’ workflow and activities. In phase 2 (system design), great emphasis was placed on creating a prototype using object‐oriented programming (OOP), unified modeling language (UML) analysis, and a prototype software model based on the information gathered in phase 1. This led to the production of a system prototype. Phase 3 (prototype implementation) involves evaluating the prototype in a focus group session and revisiting the previous two processes to finalize the prototype. Lastly, in phase 4 (system implementation and evaluation), the system will be implemented in the real world with actual users and subsequently evaluated for usability.

### 2.1 Phase 1

In the study, user‐centered approaches such as interviews and observations were employed to gather user requirements. Face‐to‐face semistructured interviews were conducted to gain a comprehensive understanding of users' needs and their expectations from the system to this end we used sample size by availability of population in this study. A total of 25 academic staff from the Urmia University of Medical Sciences, specializing in various fields of dentistry including endodontics, pedodontics, periodontics, oral medicine, orthodontics, restorative and esthetic dentistry, prosthodontics, oral and maxillofacial surgery, and oral and maxillofacial radiology, participated in the study. Two individuals from each specialized group were selected for the interviews, totaling five dentists, radiology assistants, and patients. Interviewees were carefully selected based on their experience and willingness to participate in the study. Each interview lasted between 20 and 35 min and focused on four key aspects of the system: system features, UI, public oral health education, and users' workflow (Table 2). These questions were designed by the research team and were considered crucial in the system's design process. Before conducting the interviews, permission was obtained from all participants to record their voices and transform the recordings into textual data for analysis. The interviews were conducted by one of the researchers who had prior knowledge of software development. Additionally, information regarding dentists' workflow in their private clinics and how they order exams was gathered through observation methods. This data was collected to further inform the design and development of the system.

### 2.2 Phase 2

The prototyping methodology was employed to design the required prototype based on the users' needs, as discussed in the previous section. Using this approach, the necessary prototype was quickly developed to obtain feedback and comments from users. UML was used to analyze users' needs and visualize their requirements and expectations in the use case diagram. Subsequently, the prototype was developed through OOP. The entity–relationship diagram was removed in this phase as the prototype design was completed, aligned with users' expectations and needs.

The web‐based prototype was primarily designed using hypertext preprocessor (PHP), an open‐source dynamic scripting language that offers various frameworks for programming. These frameworks enhance data management, security, and coding efficiency. A self‐made framework was utilized to develop this prototype.^17^ The architecture followed the model–view–controller pattern for better control of databases, displaying information, and efficient coding.^18^ The web application's display was achieved using the Bootstrap framework developed by Twitter, which ensures compatibility across different devices such as mobile phones, tablets, laptops, and others.^19^ To address the need for a faster system, Asynchronous JavaScript and XML technology were highly recommended and implemented to speed up the display of information and events on the client side without the need for refreshing.^20^ To ensure security, a JSON Web Token was utilized for the secure connection of the application program interface (API) in the retrofit system. Additionally, Secure Socket Layer was used for secure data transmission between the API and other devices. In anticipation of the necessary integration with mobile applications, API was applied, allowing the creation of a web application for the mobile platform.^21^ The targeted mobile operating system for this study was the Android open‐source operating system, given budget constraints, as opposed to iOS. The web‐based system was designed to run on Android mobile devices by installing a program to display information to the dentists and provide a limited set of features. The data storage utilized the MySQL database, which is free of charge. We have been considering all of the security measurement we could applied in our system to protect our data.

### 2.3 Phase 3

After designing the prototype, the implementation process for the previous phases was carried out. We invited all of the users but only 10 users were participated which they were active users in the interview section for a focus group meeting. During this session, the prototype system was presented, allowing users to interact with it, explore the system UI, and provide their feedback and comments. Following this phase, their input was carefully considered, and the users ultimately agreed that this prototype would serve as their final system. After finalizing the design, this web‐based system was hosted on a local Linux web server and showcased during the focus group session. Any issues or computer bugs that arose were thoroughly examined by the system's programmer and analyst after redesigning and reimplementing the users' requirements.

### 2.4 Phase 4

As the final stage of the system's life cycle, the last method involves implementing it in the real world with actual patient data. The initial step is to create user accounts for dentists and radiologists. Subsequently, separate training sessions are conducted for radiologists and dentists, where they are shown how to use the system in each section. Staff at the radiology center are also provided with the necessary training. On the first day, four dentists started using the system, and over the following days, the number of dentists visiting the radiology center increased to eight. Additionally, a second radiology center was introduced to the system on the first weekend (as dentists are not in the office on Thursdays, resulting in lower radiologists' workloads). Like the first center, many dental patients entered the system. Within 4 weeks, a total of four radiology centers joined the system to familiarize themselves with the workflow and data entry process.

During the process, patients associated with the dentists who are members of the system were admitted. Patients were informed about the new method and provided informed consent. After taking the radiologic images, the radiography images were sent to their respective dentists through the system. Patients were given hard‐copy photos along with usernames and passwords to access their soft‐copy images through the provided interface. Dentists, on the other hand, could access their patients' information through the system and refer them to other dentists or radiologists if needed.

In the first month of implementing the system, a formative evaluation was conducted to prevent any system failures. Subsequently, a self‐made questionnaire based on the technology acceptance model, unified theory of acceptance and use of technology, task‐technology fit, and diffusion of innovations theory was designed to evaluate the system.^22^, ^23^, ^24^, ^25^, ^26^, ^27^, ^28^, ^29^, ^30^, ^31^, ^32^ The questionnaire consists of three parts: demographic information, information technology (IT) knowledge, and system evaluation, which includes efficiency, ease of use, quality of UI, quality of communication, reliability, system satisfaction, and voluntary usage of the system (See APPENDIX A for more information). Data were analyzed using SPSS 16 and tested with backward multivariate linear regression and Pearson Correlation. The validity of the questionnaire was checked by four faculty members from the Department of Health Information Technology and two faculty members from the Dentistry Department. Additionally, the questionnaire's reliability was assessed using the test–retest method (Cronbach's *α*).^33^, ^34^ Our goal is to determine which of our system features would be helpful in system design.

## 3 RESULTS

### 3.1 Phase 1

#### 3.1.1 Interview results

The demographic information of the interviewees is presented in Table 1. The interview questions are listed in Table 2. The collected data from interviews were categorized into four main aspects. Subsequently, the results were ranked based on repetition rates (low, medium, high, and very high) and are also shown in Table 3.

**Table 1:** Four aspects of interviews questions asked from users.

Aspects	Questions
The features of this system	a. What do you expect from the system in general? b. Would you like to have collaborated with other dentists and your patients? c. Would you like to transmit only the picture or anything else? d. Would you like to run the system on smartphones? e. Would you like the system to send you notifications?
User interfaces	a. What would you like the system user interface to look like? b. Would you like a different color scheme for the system?
Public oral health education	a. Would you like the system to educate the patients in multimedia?
Dentists and radiology workflow	a. Would you like the system to help you with your tasks?

**Table 2:** Demographic information of end‐users.

Group	Number	Age (average + STD)
Dentists	20	38.69 ± 5.95
Assistants	10	31.43 ± 9.14
Radiology assistants	5	35.80 ± 4.60
Patients	5	24.20 ± 0.84
Total	40	

**Table 3:** The result of the interviews in four categories.

Aspect	Subfeature	Low (*n* < 10)	Medium (11 < *n* < 19)	High (20 < *n* < 30)	Very high (*n* > 31)
System feature	1—Speed			✓	
	2—Security			✓	
	3—Communication			✓	
	4—Notification			✓	
	5—Image processing	✓			
	6—Paramedicine information	✓			
	7—3D picture	✓			
	8—2D picture			✓	
	9—Patient access		✓		
UI	1—User‐friendly				✓
	2—Easy to use				✓
	3—Well‐designed UI			✓	
Public health	1—General training			✓	
	2—Patient's usage		✓		
	3—Helping assistant		✓		
Workflow	1—Online reservation	✓			
	2—Beneficial for dentist's workflow			✓	
	3—Beneficial for the assistant's workflow			✓	
	4—Beneficial for patient's workflow			✓	

Abbreviations: 2D, two dimensional; UI, user interface.

#### 3.1.2 Observation results

Based on the observations, the workflow in the clinics follows three main steps: (A) visiting the patients, (B) ordering radiography if necessary, and finally, (C) bringing back the radiography image/images to their dentist. In Urmia, the radiology dental sector is separated from other sectors in oral health care wards. In some clinics, imaging radiography is available only for periodical images and not for other types of dental imaging unless the principles of radiation protection are required.

### 3.2 Phase 2

**Figure 1:**
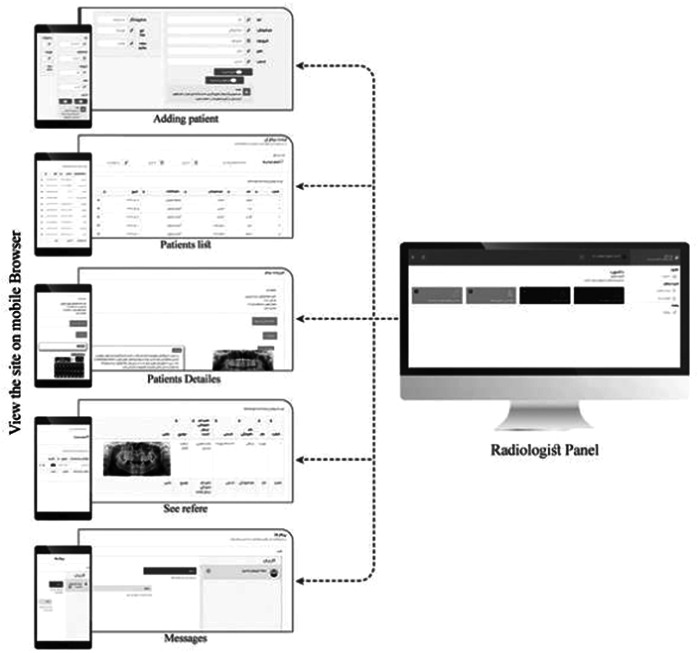
Radiologist's dashboard.

This section presents the logical and physical design of the prototype and the system. For different users, the login pages feature a dashboard that provides a brief overview of their work status, such as the number of unread messages and the number of patients. The dashboard for radiologists includes features such as adding a patient's name, last name, national code, address, doctor, birth date, phone number, disease history, and insurance code for data entry. However, only the patient's name and federal code are mandatory. When a patient is added, they are listed in the patient list, and by clicking on their name, the radiography image and its report can be added. Additionally, any referred patients are visible in the list. The message section allows radiologists to send messages to the system administrator as part of a help desk function. This dashboard is demonstrated in Figure 1.

**Figure 2:**
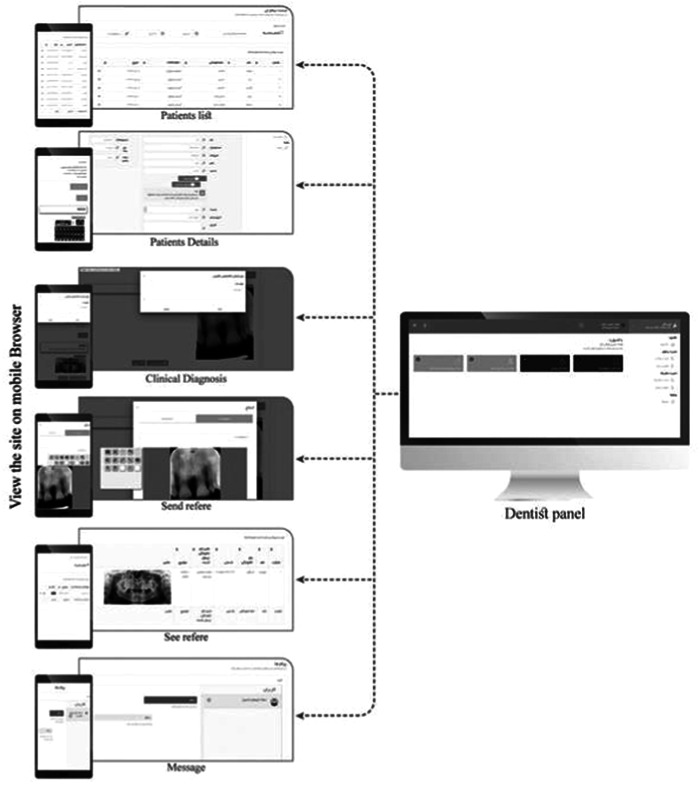
Dentist's dashboard.

**Figure 3:**
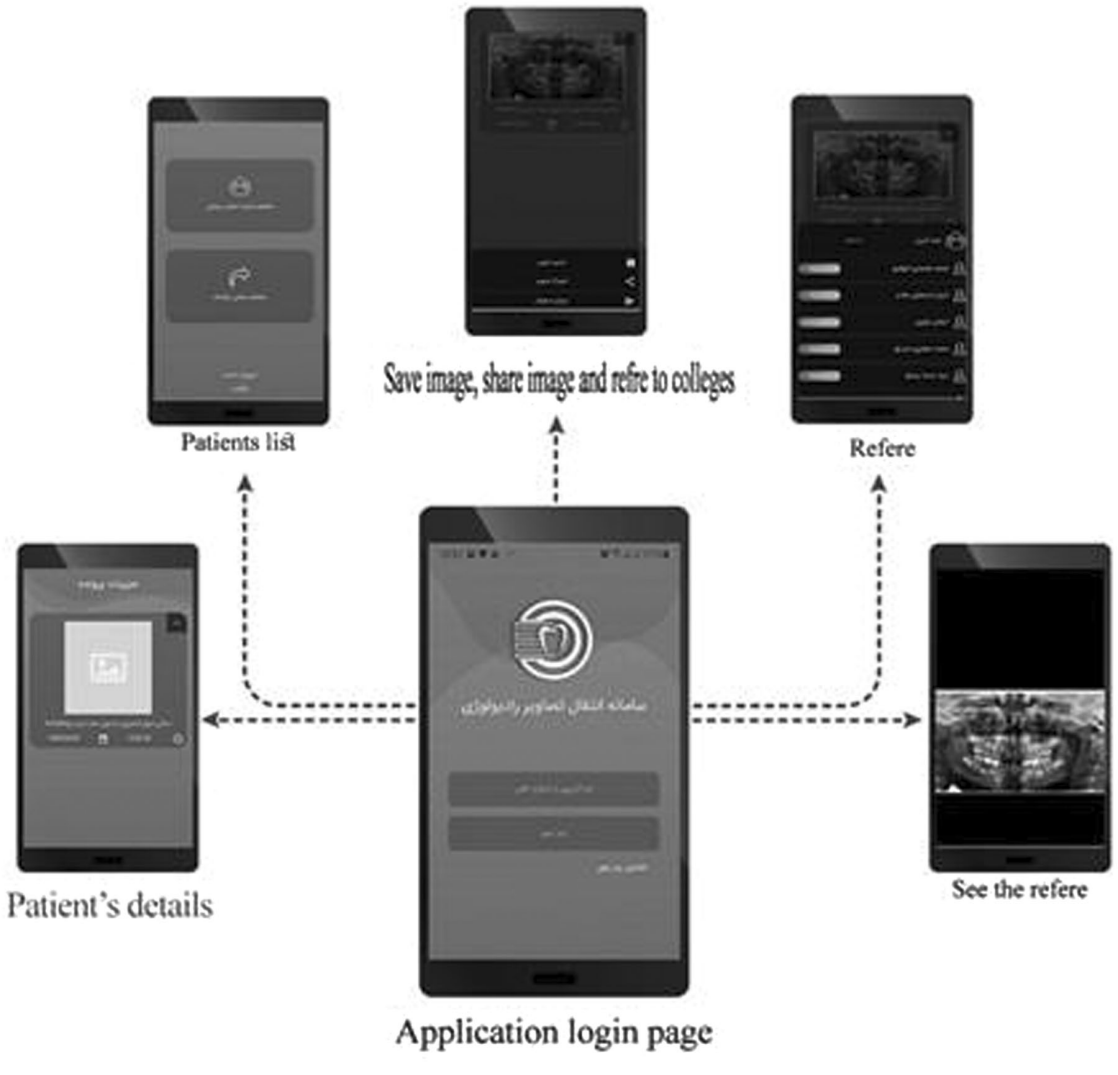
Android application.

The dentists' dashboard (demonstrated in Figure 2) includes features such as viewing their patients and their corresponding images. Additionally, dentists can send their patients' images to any colleague and access their colleagues' referred patients' radiography and reports. Furthermore, all pages are designed to be easily viewed on mobile browsers without any complications, as shown in Figure 3.

### 3.3 Phase 3

The focus group, consisting of 10 significantly active users identified during the requirement phase, carefully considered their needs and expectations before the prototype was presented. During the interaction with the system and the exploration of its UI, the participants shared their opinions. Notably, due to the easy accessibility of mobile devices, the demand for a developed mobile app was strongly recommended by a majority of the participants (*n* > 6). Additionally, there was a significant need for radiological reports and their graphical representation.

Among the other significant demands expressed by the focus group, a referral system for radiologists, the ability to add radiologic exams for patients, and the inclusion of a feature allowing users to create a mark on the photo to convey their meaning more easily to the radiologist or dentist's colleague (3 > *n* > 5) were highlighted. Subsequently, the system was modified to accommodate these requests, and the updated version was presented to another focus group. In this session, users expressed their level of satisfaction with the system's operation, data entry process, minimum data set requirements, and overall system workflow. Their feedback was carefully evaluated and taken into account by the team working on the system.

### 3.4 Phase 4

In this study, the system was implemented with the participation of 4 radiology centers, 9 dentists, and 50 patients during the first month. The questionnaire used in the study showed high internal consistency, with a Cronbach's *α* of 0.986. The correlation between the variables is presented in Table 4, and the results of the multivariate linear regression analysis are shown in Table 5.

**Table 4:** Variables correlations.

		Benefits cent	Ease of use	UI	Interaction	Reliability	Satisfaction	Voluntary	IT knowledge
Beneficent	Pearson correlation	1	0.206	0.590**	0.487*	0.543*	0.634**	0.674**	0.624**
	Significant (two‐tailed)		0.398	0.008	0.034	0.016	0.004	0.002	0.004
	*N*	19	19	19	19	19	19	19	19
Ease of use	Pearson Correlation	0.206	1	−0.174	−0.088	−0.028	−0.239	.079	−0.291
	Significant (two‐tailed)	0.398		0.282	0.720	0.910	0.138	0.749	0.068
	*N*	19	40	40	19	19	40	19	40
UI	Pearson correlation	0.590**	−0.174	1	0.666**	0.178	0.642**	0.358	0.642**
	Significant (two‐tailed)	0.008	0.282		0.002	0.465	0.000	0.132	0.000
	*N*	19	40	40	19	19	40	19	40
Interaction	Pearson correlation	0.487*	−0.088	0.666**	1	0.524*	0.884**	0.523*	0.357
	Significant (two‐tailed)	0.004	0.720	0.002		0.001	0.000	0.002	0.134
	*N*	19	19	19	19	19	19	19	19
Reliability	Pearson Correlation	0.543*	−0.028	0.178	0.524*	1	0.643**	0.664**	0.154
	Significant (two‐tailed)	0.006	0.910	0.465	0.001		0.003	0.002	0.528
	*N*	19	19	19	19	19	19	19	19
Satisfaction	Pearson correlation	0.634**	−0.239	0.642**	0.884**	0.643**	1	0.752**	0.426**
	Significant (two‐tailed)	0.004	0.138	0.00	0.000	0.003		0.000	0.006
	*N*	19	40	40	19	19	40	19	40
Voluntary	Pearson correlation	0.674**	0.079	0.358	0.523*	0.664**	0.752**	1	0.298
	Significant (two‐tailed)	0.002	0.749	0.132	0.002	0.002	0.000		0.215
	*N*	19	19	19	19	19	19	19	19
IT knowledge	Pearson correlation	0.624**	−0.291	0.642**	0.357	0.154	0.426**	0.298	1
	Significant (two‐tailed)	0.004	0.008	0.000	0.134	0.528	0.006	0.215	
	*N*	19	40	40	19	19	40	19	40

Abbreviations: IT, information technology; UI, user interface.

** Correlation is significant at the 0.05 level (two‐tailed).

* Correlation is significant at the 0.01 level (two‐tailed).

**Table 5:** Multivariate linear regression.

Dependent variable/independent variables		Unstandardized coefficients		Standardized coefficients	*t*	Significant	*R* ^2^
		*B*	Standard error	***β***			
Beneficent	UI	0.334	0.145	0.340	2.297	0.035	0.981
	Voluntary	0.599	0.135	0.657	4.443	0.000	
Ease of use	Voluntary	1.025	0.038	0.988	27.152	0.000	0.975
UI	Interaction	0.420	0.152	0.396	2.763	0.013	0.972
	Beneficent	0.611	0.146	0.600	4.192	0.001	
Interaction	Satisfaction	1.711	0.234	1.852	7.317	0.000	0.982
	Voluntary	−0.762	0.221	−0.873	−3.448	0.003	
Reliability	Voluntary	0.815	0.037	0.982	21.751	0.000	0.961
Satisfaction	Voluntary	0.564	0.053	0.597	10.655	0.000	0.996
	Interaction	0.444	0.061	0.410	7.317	0.000	
Voluntary	Ease of use	0.253	0.117	0.262	2.169	0.045	0.992
	Interaction	−0.342	0.168	−0.299	−2.032	0.049	
	Satisfaction	1.090	0.246	1.031	4.433	0.000	
IT knowledge	Beneficent	0.437	0.175	0.501	2.501	0.023	0.972
	UI	0.422	0.172	0.491	2.456	0.025	

Abbreviations: IT, information technology; UI, user interface.

A total of 40 end‐users willingly completed the questionnaire, representing different user categories: 22.5% dentists (*n* = 9), 10% radiologists (*n* = 4), 52.5% patients (*n* = 21), and 15% radiologist's assistants (*n* = 6). The participants' age had a mean of 33.30 ± 6.42 (mean ± standard deviation). In terms of education, 17.5% had a diploma (*n* = 7), 37.5% held a bachelor's degree (*n* = 15), 12.5% had a master's degree (*n* = 5), and 32.5% had a PhD (*n* = 13). Regarding their field of study, 5% were periodontists (*n* = 2), 22.5% were radiologists (*n* = 9), 5% were orthodontists (*n* = 2), 5% were oral medicine specialists (*n* = 2), 7.5% were general dentists (*n* = 3), and 55% fell into other categories (*n* = 22).

Regarding training, 50% of the users had received training for using the system (*n* = 20), while the other 50% had not engaged in any training activities (*n* = 20). Among those who were trained, 50% received in‐person training for using the system (*n* = 20). As for system usage, 90% of users used the system for less than 7 h a week (*n* = 36), while only 10% used it for more than 21 h a week (*n* = 4), mainly comprising radiologists and their assistants. In terms of device usage, 52.5% accessed the system through a PC via the website (*n* = 21), 40% used a mobile phone (*n* = 16), and 7.5% utilized a tablet (*n* = 3).

Correlations between following variables are significant (confidence interval = 95%, *p* <= 0.05):

- Beneficent with UI, interaction, reliability, satisfaction, voluntary, and IT knowledge.
- UI with beneficent, interaction, satisfaction, and IT knowledge.
- Interaction with beneficent, UI, reliability, satisfaction, and voluntary.
- Reliability with beneficent, interaction, satisfaction, and voluntary.
- Satisfaction with beneficent, UI, interaction, reliability, voluntary, and IT knowledge.
- Voluntary with beneficent, interaction, reliability, and satisfaction.

The different numbers in the correlations table indicate that some parts of the questionnaire were not answered by patients and assistants, resulting in an *N* of 19 for their responses.

## 4 DISCUSSION

In this study, a user‐centric approach was employed in designing the system, ensuring that users' opinions were taken into consideration at each stage of the system development, including analysis, development, and evaluation. Ethnographic methods such as interviews, field studies, and focus group sessions were used to assess users' needs and gather valuable feedback for system development. It worth to mention that this system was created based on the limitation of a study.^35^

The first phase was users' needs regarding their working settings in Urmia City were categorized into four general categories. The first category focused on the features of the system, including user needs related to communication with colleagues, sending images or reports to physicians, using a mobile application, and messaging functionalities. Some articles assess the usability of digital imaging or the accuracy of its technology, often focusing solely on one phase of system development for evaluation.^36^ In contrast, we made a concerted effort to include users at every stage of our study, aiming to comprehend their needs and enhance the system—an aspect not observed in our reviews.

The appearance and UI were considered as important factors in the second category. The third category encompassed the patients' economic and welfare perspective, which played a significant role in determining the system's success. To reduce project costs, free programs and methods, such as Linux Web Server, PHP programming language, Android application, and MySQL database, were utilized. Another aspect of our data gathering phase was interview. To our knowledge, there has been no report on the use of low‐cost programming methods. If programming languages have been employed, they have been integrated into the respective hospital's HIS as these were pre‐ordered PACS. Moreover, the adoption of telemedicine in rural areas has successfully reduced the costs incurred by both patients and hospitals, enabling a more active referral system.^36^

The results from dentistry faculty members at Urmia University of Medical Sciences emphasized the importance of a mobile application, having a report of images, ease of system use, and a user‐friendly interface. Speed, security, communication with others, notifications, and image processing were among the key aspects of the system that were highly solicited by users.

On the other hand, the need for paramedical information, three‐dimensional imagery, and an online booking system were considered minor requirements in users' needs analysis. The study revealed that dentists, radiologists, and their assistants were looking for a system that could provide quick results for patients without requiring extensive prior training. Thus, easy accessibility on mobile devices was a major factor contributing to the system's popularity among users. Utilizing mobile phones with new PACS systems mostly doesn't necessitate the use of any applications as they are primarily web‐based. However, we developed our own mobile application, and users expressed satisfaction with its UI and speed. In the initial years of introducing PACS, there were concerns regarding system agreements and the utilization of such systems. Additionally, once these systems were developed, the predominant concerns shifted toward the examination of new technologies. However, implementing a human‐centered approach to such systems was not very prevalent.^36^

The disadvantages of the system from the end‐users' perspective included the lack of a suitable plan in the office for quick viewing of images, the inability to upload photos from radiology center images due to high workload, and the prolonged treatment process. However, the system also brought various advantages, such as eliminating medical clips, centralized image storage, reduced chances of clips being lost or destroyed, and decreased patient exposure to radiation. Additionally, implementing this system could lead to improvements in workflow and potentially eliminate the need for physical data in the oral health sector. This system stood out from others due to the active involvement of users at all stages, the use of cost‐effective open‐source programming languages, the development of a user‐friendly mobile application, and the absence of compulsory usage.

The analysis of Pearson's correlation demonstrated that ease of use had a nominal impact on other variables. In contrast, the nature of usage—whether voluntary or compulsory—and IT knowledge exhibited significant influence over other variables. This result implies a preference among users for voluntary system usage, and it highlights how their IT knowledge significantly affects their satisfaction and ease of operation with the system.

## 5 CONCLUSION

In general, the success of the IT system in the private dental sector in Urmia City relies on providing the appropriate infrastructure and adopting a user‐centric approach with voluntary deployment of these systems. This approach can yield cost and health benefits for the stakeholders in this sector, and it has the potential to enhance the patient's treatment process by facilitating the establishment of an EHR in the private sector and promoting collaboration with the governmental sector. Consequently, the government can use this methodology to develop a successful strategy for implementing new systems in the public sector, such as the Iranian EHR. By establishing a strong relationship between the private and public sectors, the EHR can achieve a comprehensive level of integrity. As expected, when users willingly embrace a system, it leads to increased collaboration with developers and positive outcomes. Policymakers should be mindful of how their decisions impact users, as this can significantly influence the system's overall effectiveness in a positive manner.

### 5.1 Limitation

An Android mobile application was developed because there were issues with publishing the IOS app. Additionally, there was a lack of patient cooperation with the research team. Moreover, the patients were from the private sector, whereas this study was intended for an academic setting. Also, cross‐sectional study can show the potential correlation but it cannot define direct cause, therefore a cohort study and a root cause will be required to understand the root cause.

## AUTHOR CONTRIBUTIONS

**Bahlol Rahimi**: Conceptualization; data curation; formal analysis; investigation; methodology; project administration; resources; supervision; writing—original draft; writing—review and editing. **Sajjad Karimian**: Conceptualization; data curation; formal analysis; investigation; methodology; writing—original draft; writing—review and editing. **Aisan Ghaznavi**: Conceptualization; methodology; writing—original draft; writing—review and editing. **Mohammad Jafari Heydarlou**: Conceptualization; project administration; resources; writing—review and editing.

## CONFLICT OF INTEREST STATEMENT

The authors declare no conflict of interest.

## ETHICS STATEMENT

This study was extracted from a Master of Science thesis and funded partially by the Urmia University of Medical Science (Grant Number IR.UMSU.REC.1397.308), this committee reviewed the ethical and scientific aspects of this study.

## TRANSPARENCY STATEMENT

The lead author Sajjad Karimian affirms that this manuscript is an honest, accurate, and transparent account of the study being reported; that no important aspects of the study have been omitted; and that any discrepancies from the study as planned (and, if relevant, registered) have been explained.

## Data Availability

Data sharing is not applicable.

## APPENDIX A

### A.1

(I) **First section**

**General specifications**

- Level of education:
  Diploma
  Bachelor
  Masters
  Dentist
  PhD
- Field of study………….

- Gender
  Male
  Female
- Received training to use the system
  Yes
  No
- In case of a positive answer, was training downloaded from the website, or did a person train you?
  Downloaded from the website
  A person trained me
- How used the system?
  Mobile phone
  Tablet
  Website
- The average daily hours spent working with the system is…………….
- System usage during the week……………….

(II) **Second section**
  **Knowledge of information technology**
  **Table:**
  Questions	Very much (5)	Much (4)	Medium (3)	Low (2)	Very little (2)
  In general, the skills needed to work with Microsoft Office applications.					
  The level of your ability to properly install software on a computer.					
  Awareness of the benefits of using an Information system in a healthcare setting					
  The average usage of computer and mobile phone to facilitate your daily affairs					
  Daily work usage with computer and mobile at work					
  Email sent and received in the workplace.					
(III) **Third section**

**System satisfaction information**

**Table:**

No.	Questions	Very much (5)	Much (4)	Medium (3)	Low (2)	Very littls (2)
(A) *Usefulness*						
1	The system saves time in retrieving the images.					
2	The system improved access to medical resources (clinical images).					
3	The system fulfilled my clinical needs.					
4	The system is beneficial in my work.					
5	The system improved my work performance.					
6	The system improved my work efficiency.					
7	The system generally improved my work performance.					
8	The system learned how to use both usages of technology and clinical information.					
9	The system information is up to date.					
(B) *Ease of use*						
1	The system is easy to use.					
2	Learning how to use the system is easy.					
3	I get my needed information quickly.					
4	Using the system does not require much mental effort.					
5	Interactivity with the system is clear and understandable.					
6	The system's response time is fast.					
7	Using a digital system is better than a paper system.					
8	Data input is easy					
(C) *User interface* *quality*						
1	The system is attractive.					
2	I like to use the system.					
3	The system is simple and understandable.					
4	I like the system's color scheme.					
5	The system display on mobile is good.					
6	In the system, error and success messages are displayed well.					
7	The output format of the information in the system is appropriate.					
8	Appropriate colors have been used for different parts of the system.					
9	Designing system pages are simple.					
10	There is a logical connection between the pages of the system.					
(D) *System communication quality*						
1	I can communicate with other colleagues.					
2	Communication with other colleagues is easy to find.					
3	Is it possible to communicate with the site manager?					
4	Communication with the site manager is easy to find.					
5	The system search function works well.					
6	I can quickly get help when I have a problem with the system.					
(E) *Reliability*						
1	The images in the system are as good as the physical images.					
2	The images in the system are as accurate as the physical images.					
3	If I get an error or problem, I can quickly fix it.					
4	The system provides me with an excellent message to fix the error.					
5	The error message provided by the system is helpful.					
6	The system help is complete.					
7	System security is reliable.					
8	The information content of the system meets my needs.					
9	The information provided by the system is sufficient.					
(F) *System satisfaction*						
1	I feel comfortable communicating with colleagues through the system.					
2	The system is an acceptable way to receive clinical images.					
3	Using the system is the same as before					
4	I have a positive feeling about using the system.					
5	The quality of work in providing services has been improved by using the system.					
6	Using the system is essential in my job.					
7	Using the system is related to my job.					
8	Using the system does not increase my workload in the workplace.					
9	In general, I am satisfied with the system.					
(G) *Volunteering*						
1	I use this system voluntarily.					
2	Using this system, in my colleague's opinion, is required.					
3	Using this system in my department is voluntary.					
4	If the system is presented at the university, I will use it.					
5	I will also use the system to care for patients in the ordinary course of work.					
6	I will use the system in clinical and nonclinical usage.					
7	I will use the system to care for my patients continuously.					
8	I will use such a system again.					
9	I recommend using this system to others.					
10	I think others should use this system as well.					
